# Determining Risky Driving according to the Constructs of Mentalization and Personality Organization with the Modifying Role of Aggressive Driving

**Published:** 2019-10

**Authors:** Masoumeh Seydi, Isaac Rahimian Boogar, Siavash Talepasand

**Affiliations:** 1 Department of Clinical Psychology, Faculty of Psychology and Educational Sciences, Semnan University, Mahdishahr, Semnan, Iran.; 2 School of Psychology and Educational Sciences, Semnan University, Mahdishahr, Semnan, Iran.

**Keywords:** *Aggressive Driving*, *Mentalization*, *Personality Organization*, *Risky Driving*

## Abstract

**Objective** **:** This study aimed to model risky driving and predict its occurrence according to the constructs of personality organization and mentalization considering the role of aggressive driving as a mediator construct.

**Method** **:** A total of 428 individuals (219 men and 209 women) were selected using convenience sampling. The participants completed self-report questionnaires on aggressive driving, risky driving, mentalization and personality organization Also, data were analyzed using structural equating model and weighted regression.

**Results: **The results of this study showed a goof fit of the proposed structural model for predicting risky driving after some modifications (CFI = 0.95, RMSEA = 0.09). According to the results of regression weights, personality organization (regression weighted: 0.044) and aggressive driving (regression weighted: 0.98) were the strongest and mentalization (regression weighted: 0.004) was the weakest predictor of risky driving. Aggressive driving was the strongest direct predictor and personality organization the strongest indirect predictor of risky driving.

**Conclusion: **Risky driving is a function of direct and indirect personal factors. Moreover, emotional factors have a direct effect on risky driving and more substantial constructs, such as personality, have an indirect effect on risky driving.

Traffic accidents and road traffic mortalities are a managerial and social crisis worldwide. According to a WHO report, the total number of road traffic deaths plateaued at 1.35 million worldwide in 2018 (1). In its 2012 repots, WHO described Iran’s roads among the most dangerous in the world in terms of road traffic deaths. Globally, about 1.2 million people lose their lives and 20-50 million become disabled due to road traffic accidents every year (2). Part of these accidents is related to aggressive driving as a significant phenomenon in today’s world. Aggressive driving is one of the three causes of vehicular crashes and accounts for two thirds of the resulting deaths (3). Road clashes are also the second cause of mortality and morbidity in Iran; and more than 219 172 people were killed in road traffic accidents from 2005 to 2014 (2). Aggressive driving may result in impatience, anger, and reaction of other drivers. According to the Shiner (1998), both the driver and driving conditions may have a role in aggressive driving; for example, a short green light and getting stuck in traffic lead to frustration which produces anger (4). Aggressive driving is defined as “any behavior intended to physically, emotionally, or psychologically harm another within the driving environment” and encompasses a broad spectrum of behaviors such as speeding, cutting off other drivers, weaving through traffic, unsafe lane changes, disregarding traffic rules, shouting, honking, and use of high beams. Aggressive driving is an intentional action that targets another driver. In addition, driving aggression can be affected by psychological phenomena such as stress, fatigue, and impulsive behavior (5).

Road traffic accidents may be explained by personal, human, environmental, or technological factors or their combination. The exact proportion of these factors is not clear, but it seems that human factors are the leading determinant (2). 

Studies have shown that several psychiatric and psychological components can affect the occurrence or worsening of driving aggression, including alcohol consumption, psychomotor slowing, concentration impairment, desire to die, and suicidal thoughts. Personality is also among the influential factors, especially the part of personality that produces behaviors and objective reactions unconsciously. The interaction of personality with other factors can modify or amplify their roles; for instance, the interaction of alcohol consumption with other personality traits such as aggression or hostility in some drivers that makes them more prone to accidents. Also, individuals with an antisocial lifestyle, characterized by invading other people’s privacy, are usually reckless drivers. Primitive defense mechanisms as part of the impaired personality organization can augment these reactions. Intolerance of and aggression towards “power authorities” in a “high-accident” group of taxi drivers indicates that these drivers exhibit elements of conduct disorder, psychological incompatibility, and an irresponsible approach to driving and are more likely to have criminal records. In some people, especially men, adolescents, singles, unemployed, and those with low-level jobs, a car can be used as a means of expressing aggression (6). 

When Kernberg explained the development of personality dysfunction, he also described the levels of personality organization. In Kernberg’s view, the three levels of personality organization include neurotic, borderline, and psychotic levels, which can be identified using reality testing, defense mechanisms, and identity consistency. Reality testing is defined by the capacity to differentiate self from non-self, intrapsychic from external origins of perceptions and stimuli, and the capacity to maintain empathy with ordinary social criteria of reality. The use of defense mechanism and the extent to which immature (denial, splitting, and projection), mature (humor, sublimation), or neurotic (idealization, reaction formation, and undoing) mechanisms are applied also help determine the level of personality organization. Identity diffusion refers to behavioral and psychological indicators resulting from lack of an integrated identity, especially an integrated self. Individuals with a neurotic personality organization have intact reality testing, a consistent identity, and more mature defense mechanisms such as undoing, reaction formation, and suppression. People with the borderline level of personality organization have generally intact reality testing, immature defense mechanisms, and identity diffusion. In the psychotic levels, reality testing is severely compromised, there is an inconsistent sense of self, and immature defenses are utilized (7). According to Kernberg, individuals with a strong ego suffer from less impairment (8, 9). Lower levels of personality organization are associated with more severe clinical manifestations and poorer psychosocial function. Among different dimensions of personality organization, identity diffusion and dominant use of primitive defense mechanisms are markedly associated with poor functionality (10). Among personality traits, extraversion and neuroticism have a positive correlation and agreeableness, and conscientiousness have a negative correlation with accident involvement. A strong positive correlation was found between extraversion and risky driving (11). Several studies have shown a strong correlation between higher levels of extraversion and vehicular clashes and road traffic death and also between neuroticism and dangerous driving behaviors. Individuals with psychoticism are inattentive to others’ feelings and ignore traffic and social rules and norms. Lev et al (2008) found that traffic offenders were more extraverted. Another study showed that neurotic people exhibit more mood swing and irrationality as a result of anxiety, restlessness, depression, and tension, which can lead to risky driving (12). However, anger is another factor that can modify personality factors. Anger has a mediating role and personality characteristics are more important factors that can result in road accidents through their effect on provoking anger in the driver. State-train anger theory explains that state anger is directly dependent on trait anger, and individuals with higher trait anger experience higher state anger as well. State anger manifests itself in behaviors like speeding, acceleration, and red-light violations (3). High-anger drivers are more likely to be involved in aggressive driving, weaving through traffic, aggression, and traffic accidents (13).

A study was conducted in 2014 to evaluate driving-related issues in four levels of aggressive violations, ordinary violations, errors, and lapses. The participants completed the Personality Inventory for DSM-5, which indexes 5 broad personality domains (antagonism, detachment, disinhibition, negative affectivity, and psychoticism) and 25 specific trait facets. The results showed that the personality domains of antagonism and negative affectivity were the best predictors of aggressive and ordinary violations, while negative affectivity was the best predictor of errors and lapses. A more analysis of trait facets revealed that antagonism was the best predictor of aggression, risk-taking, irresponsibility, and insecurity. In particular, traffic violations were associated with higher level of thrill-seeking, impulsivity, disinhibition, and negative urgency. The results of this study showed that extreme social deviance markedly predicted crash rates directly and indirectly through violations (14).

Studies investigating the relationship between narcissism and risky driving suggest that drivers with higher levels of narcissism are more likely to be involved in risky driving and carry a higher risk of death due to road traffic accidents (15).

A study by Endriulaitiene et al in 2018 showed a significant positive correlation between attitude towards risky driving and personality traits. In men, Machiavellianism had a significant correlation with subscales of attitudes towards speeding, showing off driving skills to others, joyriding, and violating traffic rules. Psychopathy was related to the majority of scales of risky driving, except for attitude toward showing off driving skills. Male narcissism had a less significant correlation with attitude towards risky driving. Moreover, there was a weak correlation between narcissism and attitudes towards speeding, joyriding, and showing off driving skills. In females, psychopathy was positively associated with all types of risky driving attitudes. Machiavellianism was correlated with all scales of attitude towards risky driving, except for attitude towards traffic rules violation. Narcissism had a positive correlation with attitude towards violation of traffic rules and speeding (16). 

Havârneanu et al found that environmental factors such as conflicts with clients, conflicts with peers, running a red light, etc., increased the rate of risky driving among taxi drivers, posing a threat to them and other drivers. The results of this study showed that experienced drivers underestimated traffic dangers, resulting in increased rates of road traffic accidents and risky driving (17).

However, the role of more important factors should not be neglected. Environmental factors cannot explain how personality and individual problems in different levels boost risky behaviors, or how people justify these behaviors or are not influenced by emotional factors like anger. Although road and vehicle characteristics and environmental factors have a significant share in traffic accidents, risky driving is an entirely human action and its determinants are also human factors. Several studies have addressed personality components and mentalization and their effects on driving aggression, and the effect of aggressive driving on risky driving. It seems that these factors can be summarized in a causal model to explain risky driving. In fact, although a number of investigations were conducted to explain the effective factors that have explained risky driving, there is a considerable gap in the integrity of causal factors and their share in risky driving. Therefore, this study was conducted to test the following model for defining risky driving based on experimental evidence obtained from previous studies.

## Materials and Methods

A questionnaire-based survey was applied in this cross sectional study.

***Population, Samples and Sampling Method***

The research population was all intra city light vehicle drivers in Tehran who had level 2 (B) and 3 (C) driver’s license. Since the research population was very extensive, convenience sampling was done on individuals of legal driving age who were available and willing to participate in the study, and 428 drivers were selected. The inclusion criteria were as follow: being at least 20 years old, holding a level 2 (B) or 3 (C) driver’s license, at least 2 years of intercity driving experience, and living in Tehran. Exclusion criteria were as follow: cognitive ability to understand the traffic signs and questions in the test and being a truck driver and driving on intercity roads, which were inquired through asking direct questions about the type of the vehicle and driving routes. 

***Measurement Tools***

***Personality Organization***

This is a 57-item self-report questionnaire in a Likert Scale and encompasses three subscales of impaired reality testing (20 items), identity diffusion (21 items), and primitive defense mechanisms (16 items). This questionnaire is developed based on the Kernberg’s definition of personality organization. A higher score in each subscale indicates more impairment in that component. Psychometric analysis of the questionnaire in a healthy population has shown that all three subscales enjoy an acceptable internal consistency (α > 0.81) and short-term test-retest reliability (18), and in another study, the reliability index was reported to be from 0.62 to 0.98 (19). In this study, the Cronbach’s alpha coefficient was 0.92 for the whole scale and 0.92 and 0.82 for the subscales of identity diffusion and reality testing, respectively. The Spearman-Brown coefficient was estimated in a range of 0.87-0.90. 

***Mentalization***

The 28-item mentalization scale (MentS) developed by Dimitrijević in 2017 was applied to measure mentalization. This scale includes the subscales of personal desires, needs, feelings, beliefs, and reasons. Psychometric analysis of the scale showed its high internal consistency in the community and in a clinical sample (0.84 and 0.75, respectively) (19). In this study, the Cronbach’s alpha coefficient was 0.78 for the whole scale and 0.83, 0.81, and 0.62 for the subscales of personal desires, needs, and feelings, respectively. The items of the MentS are designed in a 5-point Likert scale, and a higher score in the whole scale or each subscale indicates more extensive use of mentalization. 

***Aggressive Driving***

The Aggressive Driving Scale is a 24-item self-report measure developed by Fenske and Krahe in 2002 and evaluates aggressive driving in 3 subscales. A 5-point response format accompanies each item (20). In this study, the Cronbach’s alpha coefficient was 0.86 for the whole scale and 0.69, 0.74, and 0.61 for the subscales of hostility, aggression, and anger, respectively. A higher scale is indicative of a more aggressive driving behavior. 

***Risky Driving***

This questionnaire is a 24-item self-report scale developed by Iversen et al in 2004 and measures 3 subscales of negative driving (cognitive/emotional), risky driving, and anger driving using a 5-point Likert scale from 0 (never) to 4 (very often) (21). In this study, the Cronbach’s alpha coefficient was 0.90 for the whole scale and 0.81, 0.76, and 0.82 for the subscales of negative driving, anger driving, and risky driving, respectively. A higher score indicates more risky driving behavior. 

***Data Collection Method***

Eligible drivers were selected from taxi stations, drivers around Tehran who had referred to Tehran Traffic Police for license driving renewal and those working in taxi service agencies. The participants were provided with the translated versions of the above scales and requested to complete all forms. Inclusion criteria were applied to select the participants and informed consent was obtained from them prior to the study. Each questionnaire was completed in 20-30 minutes. The participants were allowed to complete the questionnaires and return them to the researcher at their convenience if they became tired during the process. 

***Data Analysis***

In addition to descriptive tables, modeling according to structural equations was one for data analysis and fitting of the model.

## Results

Table 1 shows the age, education level, and sex distribution of the participants. The mean age of the participants was 36.64±8.27 years, and 51.6% of them were male. Most of the drivers had a master’s degree followed by a bachelor’s degree. 

**Figure 1:**
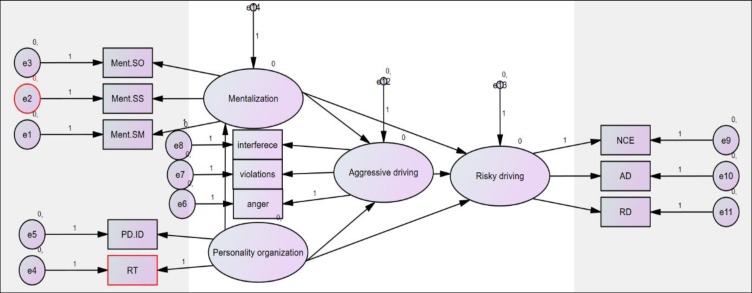
Conceptual Model for Explaining Risky Driving Based on Mentalization, Personality Organization, and Aggressive Driving

Table 2 presents descriptive data of the variables, including mean and standard deviation, and Figure 1 demonstrates the proposed causal model for explaining risky driving. The results of the analysis of this model are depicted in Table 3 and Table 4. According to Table 3, goodness-of-fit test of the initial model showed that it required some modifications (RMSEA = 0.145, CFI = 0.865, NFI = 0.853). These modifications are covariance of e2, e5, e2, and personality organization. 

After modifications were applied, all parameters improved, RMSEA decreased to 0.09, and CFI and NFI increased to 0.951 and 0.938, respectively. Moreover, marked improvements were also observed in other goodness-of-fit indexes such as IFI, PNFI, and PGFI and chi2-index decreased. However, the values of other chi2-related indexes were still far from optimal values. Since this index is a function of the sample size and reflects the smallest differences in comparison of the data and the expected model, it was recommended that other goodness-of-fit indexes be used for analysis, which confirmed the model after modification. In particular, CFI, RMSE, and other indexes showed that the variables, in the mentioned order, could correctly predict risky driving. 

The results of Table 4 revealed that personality organization was an effective and significant predictor of aggressive driving, and aggressive driving was a positive predictor of risky driving. Moreover, personality organization was a direct predictor of risky driving. According to Table 4, this variable could significantly predict risky driving both alone and in an indirect manner. Among three factors, including aggressive driving, mentalization, and personality organization, aggressive driving was the strongest and mentalization was the weakest predictor of risky driving. Also, after aggressive driving, personality organization was another predictor of risky driving. Indirect and direct coefficients presented in Table 4 also suggest that mentalization lacked any indirect effects on risky driving, while the indirect effect of personality organization on risky driving was more pronounced than its direct effect. Table 5 presents the correlation coefficients between components of predictor variables and risky driving components. 

**Figure 2:**
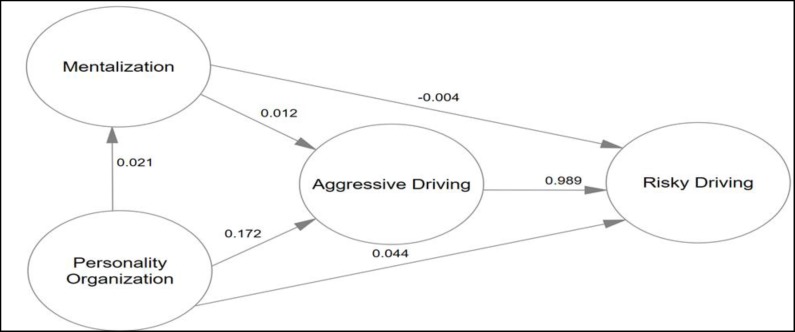
Structural Coefficient for Mentalization, Personality Organization and Aggressive Driving to Predict Risky Driving based on Concept Model

This coefficient has been represented in figure 2.

***Recommendations***

Travels and journeys are expected to increase with an increase in the use of personal cars. Therefore, the results of this study can be used to expand educational programs aiming at empowerment. More sophisticated models can be developed to describe the determinants of risky driving through conducting systematic reviews. 

## Discussion

According to the results, personality organization is an effective construct that can explain risky driving both directly and through its effect on aggressive driving. However, personality, as the self-integrity of people, has a significant share in behavioral manifestations. If thought, feeling, and behavior are accepted as personality components and considering personality stability in different situations, it can be postulated how driving can be influenced by this construct, as Kernberg clearly states how personality levels are affected by its components and direct human behaviors. People with weak egos, primitive defense mechanisms, and high levels of personality diffusion exhibit more disorganized and unpredictable behaviors and the weaker is their ego, the weaker they are in controlling impulses like anger. This is the reason why any increase in the severity of the symptoms defining borderline or psychotic personality in the levels of personality organization is associated with increased risk of aggressive driving and risky driving is higher. According to Kernberg, defense mechanisms are one of the components of personality organization defined as a mental function protecting the person against stress. Although defense mechanisms have differences in details, they share two factors: denial or reality distortion and unconscious activity (22). A study by Zoccali et al showed a correlation between defense mechanisms and anger expression and experience, as people who used more mature defense mechanisms could control their anger and prevent its expression better and vice versa (23). Mature defense mechanisms facilitate anger experience and decrease its expression, while immature mechanisms prevent anger experience and increase their expression. Moreover, the results of a study (24) showed that primitive defense mechanisms could predict personality disorders such as borderline and antisocial personality disorders in which inability to control emotions, including anger, is a prominent feature. Therefore, it could be predicted that people suffering from these disorders develop emotional reaction when exposed to frustrating situations during driving and embark on aggressive and risky driving. 

Several studies have assessed the relationship between personality and aggressive and risky driving. According to a study in 2006, participants with higher levels of extraversion and neurosis were more prone to exhibiting dangerous driving behaviors like speed limit violation, illegal overtakes, and traffic accidents (12). Tao et al reported the same results (11). Moreover, Jovanovic et al found that driving-related anger was a mediator between personality traits and aggressive driving (13).

The results of the present study showed that mentalization was an effective predictor of aggressive driving. Metallization is defined as “the mental process by which an individual implicitly and explicitly interprets the actions of himself and others as meaningful on the basis of intentional mental states such as personal desires, needs, feelings, beliefs, and reasons” (24, 25). This definition is semantically consistent and even overlaps with constructs such as empathy, social cognition, emotional quotient (EQ), and theory of mind (26). However, in contrast to empathy and social cognition, metallization also includes self-reflection, which goes beyond perceiving and understanding emotions (the realm of EI) or attributing intentions, thoughts, and beliefs, calling on various inner states and processes to interpret manifest behavior. It could be stated that mentalization has a close relationship with attachment, and securely attached persons exhibit a superior capacity to mentalize compared to those with insecure attachment. Moreover, the indicators of a well-developed metallization capacity include awareness of the nature of mental states, explicit efforts to identify mental states underlying particular behavior, recognition of developmental aspects of mental states, and awareness of mental states in relation to the interviewer (27). In this model, in this study, the personality organization explained the high proportion of the variance, showing that mentalization presented low regression coefficient to predict risky driving, and this was while the correlation matrix in Table 5 shows a high correlation between mentalization and risky driving.

The presence of some effective factors such as personality organization may be covered for the share of mentalization to some extent, because everything that finds an opportunity to manifest due to mentalization can also be affected by personality organization. Since personality organization explains the share of defense mechanisms in aggressive and risky driving, it seems that the share of this factor is explained by another more important factor encompassing it. Mentalization weakens in people with a pathological personality and loses its function, because the two are highly correlated with each other and have a common variance. Similarly, Diamond (2014) and Fonagy (2009) investigated the relationship between personality organization and mentalization and found that personality pathology was correlated with mentalization failure. In other studies by Alavi et al on Iranian drivers, neuroticism dimension was recognized as a positive predictor of traffic violence, and based on this study, depression and anxiety could increase the probability of accident occurrence (28, 29).

Finally, it was observed that aggressive driving led to risky driving. This is not the first report of the effect of emotion of anger on behavior expression and occurrence of risky situations and a large body of experimental evidence supports this relationship. The effect of unconscious impulses on self-destruction and self-punishment due to some mistakes in life and a feeling of guilt can expose the person to the situation. Frustration is defined as a reaction to a blocking of a desirable goal that can be objective or subjective; for example, a person believes that they are reaching their desirable goal and anticipates its pleasures, or it may manifest in the person’s behavior (30). Exposure to frustrating events is an inseparable part of life and it seems that humans have developed an innate preparedness for confronting these events. Aggression is one of the reactions to frustrating situations that can be physical, verbal, or in the form of disobedience. It is rooted in one of the most fundamental human desires, eg, anger (31). Sensation seeking and impulsivity lead to risky and aggressive behaviors; thus, these two construct are strongly related (32).

Driving is one of the situations where aggressive behaviors may occur, because it has several key anger-provoking components, including external control exerted through traffic codes, driving routes, and driving rules (speed limit, prohibition of cellular phone use while driving, use of seat belts, prompt attention to traffic lights or police commands, etc.), situation control, traffic, length of red lights, and personality characteristics of the driver (internal control, sensation-seeking, psychological disorders) (4). Therefore, driving is a condition in which the emotion of anger can overtake from the lowest to the highest level for different reasons, including internal and external control. Similarly, Nesbit et al (2007) showed a direct relationship between anger and risky driving that was described by the state-trait anger. The state-trait anger was conceptualized by Spielberger who differentiated state anger from trait anger. He defined trait anger as a chronic tendency and described state anger as a measure of expressing anger (3). Moreover, according to Deffenbacher et al (2000), high-anger drivers are more likely to be involved in aggressive driving behaviors, dangerous car maneuvers, violence in traffic, and traffic accidents. 

**Table 1:** Distribution of Demographic Characteristics for Participants

		**Mean/n**	**Std/%**
Age		36.64	8.27
Educations	Below diploma	17	0.4
	Diploma	56	13.3
	Associate degree	22	5.2
	Bachelor	105	24.9
	Master	155	36.7
	PhD	67	15.9
	missing	6	
sex	Male	219	51.6
	Female	209	48.4
	Total	428	100

**Table 2:** Descriptive Statistics for All Variables in the Structural Model to Predict Risky Driving

	**Mean**	**Std. Deviation**
interference	17.1262	4.02719
violations	14.6355	4.06883
anger	8.4136	2.42344
NCE	18.2500	5.36540
AD	10.1519	2.89194
RD	17.1612	4.72471
PD.ID	63.9486	19.96829
RT	21.4533	8.70571
Ment.SO	39.6939	5.70979
Ment.SS	19.2780	6.64205
Ment.SM	36.3738	5.12380

**Table 3:** Goodness of Fit Indexes for Basic Model and Modified Model to Predict Risky Driving

**The goodness of fit indexes**	$X^{2}$	$\frac{\chi 2}{\mathrm{df}}$	${\mathrm{RMSEA}}^{1}$	${\mathrm{CFI}}^{2}$	${\mathrm{NFI}}^{3}$	${\mathrm{IFI}}^{4}$	${\mathrm{PNFI}}^{5}$	${\mathrm{PGFI}}^{6}$
Expected value	p> 0.05	<3	<0.1	>0.95	>0.90	>0.90	>0.50	>0.50
Basic model	<0.0001	9.961	0.145	0.865	0.853	0.866	0.590	0.598
Modified model	<0.0001	4.446	0.090	0.951	0.938	0.951	0.622	0.614

1. Root mean square error of approximation

2. Comparative fit index

3. Goodness of fit index

4. Normed fit index

5. Incremental fit Index

6. Parsimonious normed fit index

7. Parsimonious goodness-of-fit index

**Table 4:** Unstandardized and Standardized Regression Weight to Predict Risky Driving

Predictive	Predicted	Regression weighted		P value	Indirect standardized coefficient
		unstandardized	standardized		
Personality organization…>	Metallization	0.100	0.021**	0.001	
Metallization…>	Aggressive driving	0.001	0.012	0.907	
Personality organization…>	Aggressive driving	0.058	0.172***	<0.0001	
Aggressive driving…>	Risky driving	2.57	0.989***	<0.0001	
Metallization	Risky driving	-0.001	-0.004	0.907	0.002
Personality organization…>	Risky driving	0.038	0.044*	0.029	0.149**

**Table 5:** Coefficients Correlation between Components of Predictor Variables and Risky Driving Components

		Ment.SO^4^	Ment.SS^5^	Ment.SM^6^	PD.ID^7^	RT^8^	interference	violations	anger	NCE	AD
NCE^1^	a*	0.138	0.137	0.184	0.253	0.088	0.697	0.651	0.555	1	
	b**	0.004	0.004	0.000	0.000	0.069	0.000	0.000	0.000		
AD^2^	a*	0.066	0.105	0.096	0.214	0.085	0.705	0.673	0.473	0.648	1
	b**	0.173	0.030	0.047	0.000	0.078	0.000	0.000	0.000	0.000	
RD^3^	a*	0.007	0.114	0.001	0.203	0.124	0.515	0.582	0.656	0.598	0.538
	b**	0.893	0.019	0.981	0.000	0.010	0.000	0.000	0.000	0.000	0.000

1 . negative Cognitive Emotional Driving

2. Aggressive Driving

3. Risky Driving

4. Other-related Mentalization

5. Self-Related Mentalization

6. Motivation to Mentalize

7. Primary Defense with Identity Diffusion

8. Reality Testing

* correlation coefficient

** P value

## Limitation

A limitation of the present study was lack of access to random samples, which was not practically possible due to the extensively large target population. However, the researchers minimized the effect of this limitation by increasing the sample size, homogenizing the sex distribution of the samples, and selecting drivers with different education levels. Also, methodology of the study did not allow the investigators to find a causality model for risky driving. However, this limitation was controlled by statistical methods and estimating the contribution of each variable to risky driving by SEM.

## Conclusion

According to the goodness-of-fit of the model, personality organization was the strongest construct explaining aggressive and risky behaviors, even in driving, and other constructs were influenced by this psychological integrity.
